# The Multifarious Link between Cytochrome P450s and Cancer

**DOI:** 10.1155/2020/3028387

**Published:** 2020-01-03

**Authors:** Abdullah M. Alzahrani, Peramaiyan Rajendran

**Affiliations:** Department of Biological Sciences, College of Science, King Faisal University, Hofouf, 31982, Saudi Arabia

## Abstract

Cancer is a leading cause of death worldwide. Cytochrome P450s (P450s) play an important role in the metabolism of endogenous as well as exogenous substances, especially drugs. Moreover, many P450s can serve as targets for disease therapy. Increasing reports of epidemiological, diagnostic, and clinical research indicate that P450s are enzymes that play a major part in the formation of cancer, prevention, and metastasis. The purposes of this review are to shed light on the current state of knowledge about the cancer molecular mechanism involving P450s and to summarize the link between the cancer effects and the participation of P450s.

## 1. Introduction

Functional genomics, transcriptomics, and proteomics have improved the speed of discovery in scientific research by adding a genome-wide perspective. In the majority of these studies, analyses of individual gene groupings and entire genomes have demonstrated that the extension of multigene families through gene duplication is a critical and repeating phenomenon in the advancement of new gene capacity and increasingly complex living things. As early as the 1930s, researchers hypothesized that repetitive duplicates of existing genes would be under decreased specific pressure and could change and ultimately develop new functions [1, 2]. Later, researchers would contend that gene and genome duplications were an essential, although not the only, mechanism by which new gene capacity could emerge. Because of repeated duplications throughout history, multigene super families, such as the serine proteases and the protein kinases, have come to represent an important portion of protein coding groupings in the genomes of complex life forms [3, 4]. The cytochrome P450s (P450s) comprise various hemoproteins and are one of the largest and most functionally versatile superfamilies. The functional range of P450 activity is remarkable from microscopic organisms to humans [5].

The history of “P450 investigation” began in the early 1950s, and originally, it was thought that “P450” was a solitary cytochrome that was present only in the liver and that its role was to process drugs and other synthetic exogenous substances. These protein interactions have been studied, and it was suggested that they were of clinical importance in medicine and treatment [6, 7]. With the explosion of molecular science in the 1980s, P450 genes were revealed to exist in practically all animals, from prokaryotes to rodents and humans, and the amino acid sequences prompted the main proposal of a transformative disparity-dependent gene classification system. This hypothesis proposed that all P450 genes arose today from a lonely precursor, most likely more than three billion years ago. Six vertebrates, namely, rodents, mice, humans, rabbits, dairy cattle and chickens, and yeast and *Pseudomonas putida* acquired the 30 genes originally announced in 1987. A quarter of a century later, the group had expanded to an Internet nomenclature that aggregates 18,687 protein-coding genes called P450s with putative tasks beginning in 2012 [5, 8]. P450s are helpfully organized into families and subfamilies in view of the percent amino acid similarity. Proteins sharing more than or approximately 40% identity are relegated to a specific family assigned by an Arabic numeral, while those sharing more than or approximately 55% identity make up a specific subfamily assigned by a letter. For instance, both sterol 27-hydroxylase and 25-hydroxy-D 1*α*-hydroxylase are assigned to the CYP27 family since they share more than 40% sequence identity [8, 9]. The human genome thus contains 18 P450 families, divided into 41 protein-coding subfamilies encoding 57 genes [10].

**Figure 1:**
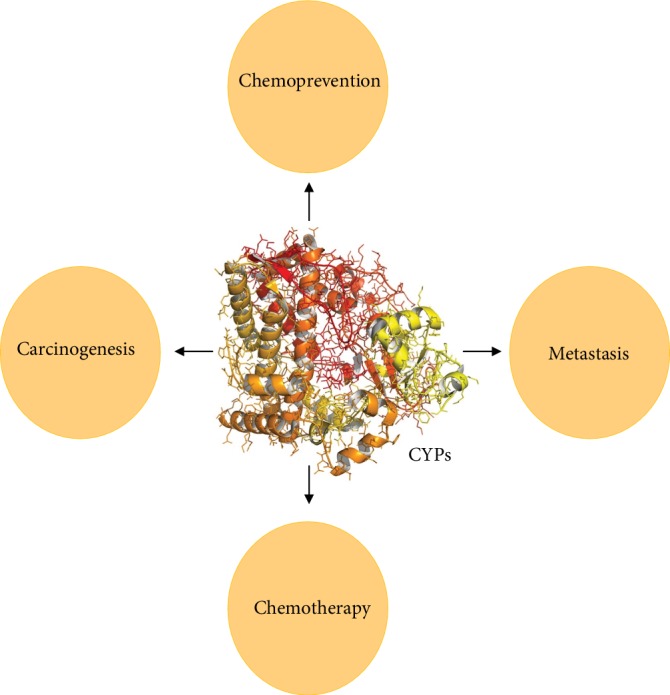
The potential role of P450 family proteins in cancer.

A growing amount of studies on P450s showed throughout the 1970s and 1980s that they were present in many species with numerous apparently unrelated life processes, including in crops; therefore, P450s were suggested to be significant upstream molecules in the synthesis and degradation of nearly all nonprotein ligands binding to receptors or activating second-messenger pathways governing growth, homeostasis, apoptosis, and neuroendocrine and differentiation functions [11]. As a rule, therapeutically relevant P450s affect compounds that are similar to essential endogenous substrates and can affect their functions. Numerous human P450 drugs are used to treat human maladies. Others are important for amalgamation of endogenous substances fundamental for human physiology. In a few occurrences, changes in explicit P450s can influence various processes and lead to serious human diseases [12]. Cancer is a complex disease mediated by many molecular and cellular processes. Cancer constitutes one of the world's major causes of death [13]. A global metabolic network expressed in many species, including phase I xenobiotic enzymes, CYPs, and phase II xenobiotic-conjugating enzymes, also uncovered a metabolic pathway that was discovered to bioactivate chemicals and cause cancer. Many researchers have found that P450s (1A1, 1A2, 1B1, 2A6, 2A13, 2E1, and 3A4) play a role in the activation of different carcinogenic compounds in the environment, including tobacco-related nitrosamines and polycyclic aromatic hydrocarbons (PAHs) [14–16]. Electrophilic intermediates from various tobacco-related nitrosamines and PAHs can form covalent bonds on DNA nucleotides, particularly protooncogenes or tumor suppressors, with nucleophilic areas. With the lack of precise DNA repair of adducts, these chemical changes can lead to changes in the encoded genes, which in turn can start the shift from an ordinary cell to a cancer cell [17]. P450s are the most important enzymes that catalyze reactions related to antineoplastic agents. The use of cytotoxic antineoplastic agents in chemotherapy remains a key part of the management of malignant tumors. The CYP2A, CYP2B, CYP2C, CYP2D, and CYP3A subfamilies are mainly responsible for the metabolism of anticancer drugs. One of the most important problems in the treatment of intracranial tumors is chemoresistance [18, 19]. Increased concentrations of P450s may result in intracellular drug inactivation. It has also been suggested that local expression of P450s in tumors is essential for cancer management because P450s expressed in tumors may be involved in chemotherapeutic drug activation and/or inactivation [20]. P450s may be present in tumor cells as part of a pleiotropic reaction to tumor growth. Moreover, they could partially weaken the production and migration of cells. For instance, CYP2W1, CYP1B1, CYP2C9, CYP2C8, CYP2J2, and CYP4A have been linked to tumor-specific expression. These findings have led to the identification of the part played by P450s in cancer growth and tumor formation. For that reason, P450s have a crucial role in cancer formation, chemoprevention, metastasis, and chemotherapy (Figure 1). The aim of this review was to summarize the detailed impact and involvement of P450s in human cancer.

## 2. Role of P450s in Cancer Formation

As people reach their 6th decade, they face an exponentially expanded risk of developing cancer. Approximately 5% of human malignant growths are caused by infections, 5% by radiation, and 90% by chemicals. Of these, an expected 30% are caused by the utilization of tobacco and the rest by synthetic substances related to diet, lifestyle, and the environment [21]. The significance of these substances in the etiology of malignant growth is reflected by the finding that up to 8% of cases of every single human disease are related to synthetic factors [22]. Synthetic cancer-causing agents, or their metabolites, are exceptionally receptive electrophiles, which have electron-insufficient molecules that can respond to nucleophilic, electron-rich regions in the cell [23]. Deoxyribonucleic acid (DNA), specifically, comprises a variety of nucleophilic foci at which these DNA-damaging agents can form DNA adducts through at least one covalent bond [24, 25]. CYP-mediated reactions can be used to examine the potential of chemicals to be activated to form reactive species, leading to the formation of covalent DNA adducts [26–28]. In the large group of enzymes involved in the metabolism of carcinogens, P450s are the most important enzymes in the metabolism of PAHs [29–33].

### 2.1. Xenobiotic Metabolism

Procarcinogens are extremely hydrophobic compounds found in “xenobiotics.” Therefore, these procarcinogens are substrates for some P450 family 1 isoforms (CYP1A1, CYP1A2, and CYP1B1) [16, 34, 35]. The substrate-inducible CYP1A1 and CYP1A2 are responsible for xenobiotic metabolism, such as polycyclic aromatic hydrocarbon metabolism [36–40]. In this group, charred food and cigarette smoke catalyze N-oxidation of carcinogenic aromatic and heterocyclic amines [41–43]. The enzymes are selective for planar molecules, which are carcinogens, as substrates. The substrates are then oxygenated in conformationally delayed positions that result in the formation of highly reactive epoxides. These peptides that are not easily detoxified by glutathione transferase, epoxide hydrolase, and other detoxification enzymes are cytosolic (steroid-like) receptors. The AhR receptor [44] regulates peptides that bond with carcinogens and other planar molecules, resulting in the increased production of P450s, AhR protein, and other enzymes through genomic depression. This change also activates the protein kinase C cascade [45]. While carcinogenic chemicals are activated metabolically by P450 family 1 members, some small molecules, such as nitrosamines, are activated by CYP2E1. The majority of xenobiotic procarcinogens are characterized as highly hydrophobic. In this respect, these procarcinogens act as substrates for the enzymes CYP1A1, CYP1A2, and CYP1B1 [46]. In addition, the CYP1A1 and CYP1B1 enzymes metabolize PAHs that are exceptionally hydrophobic as well as polyhalogenated aromatic hydrocarbons (PHAHs) [47] (Table 1). For effective metabolism, at least two nearby unreplaced positions in the aromatic ring of PHAHs are essential. Those PHAHs that do not have this feature are metabolized very slowly, resulting in a chemical half-life varying from a few weeks to months and even years at times. The coplanar PHAHs and PAHs that degrade slowly are effective inducers of CYP1 member enzymes due to their strong attraction to the AhR [48]. AhR regulates the three CYP1 member genes, which in turn are activated due to the binding of coplanar PHAHs and PAHs. AhR can react with estrogen receptor-*α* [49], nuclear factor *κ*B (NF-*κ*B) [50], and retinoblastoma protein 1 (RB1) [51]. This in turn leads to gene transcription that participates in growth, apoptosis, and the cell cycle. AhR can therefore be considered a xenobiotic-metabolizing enzyme (XME) receptor that promotes CYP1 member enzymes that metabolize procarcinogens. Moreover, AhR may also promote environmental carcinogenesis by changing cell-cycle functions, including apoptosis, without relying on CYP-mediated ROMs [34]. The CYP1 member enzymes metabolize an endogenous ligand for the identified AhR [52].

#### 2.1.1. P450s and 7,12-dimethylbenz[a]anthracene (DMBA)

**Figure 2:**
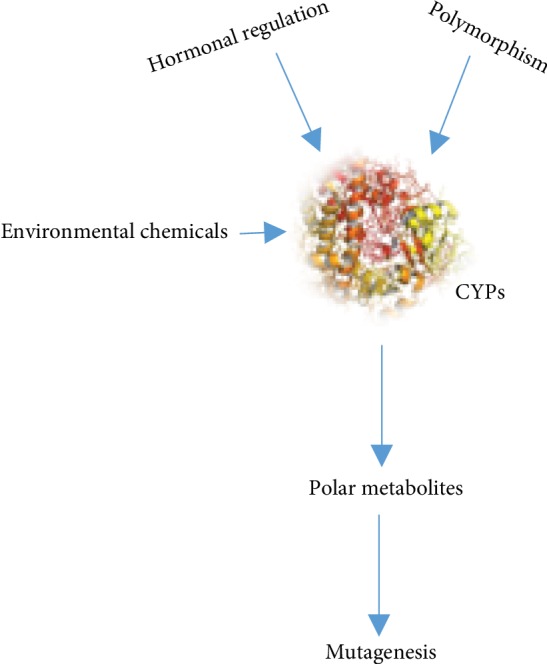
P450s in cancer formation.

The chemical 7,12-dimethylbenz[a]anthracene (DMBA), which is commonly used as a model chemical carcinogen, is a PAH. The compound has been applied in a rat mammary tumor model [30] and is the most studied PAH apart from benzo[a]pyrene (B(a)P). In vivo, CYP1B1, and not CYP1A1, was shown to be the main enzyme responsible for the metabolic activation of DMBA when exposed to carcinogenic metabolites [53]. While 3,4 diol-DMBA, 7 1,2-epoxide-3,4-diol-DMBA, and -OHM-12DMBA have all been proven to show genotoxic effects, the latter was found to be the ultimate carcinogen [54]. Substantial amounts of deoxyadenosine and deoxyguanosine adducts have been shown to be induced by the benzylic carbon of 1,2-epoxide-3,4-diol-DMBA [54]. In some cases, researchers have found minor adducts with the amino group of the 7-position of deoxyguanosine and deoxycytidine [55]. Some researchers believe that DMBA is a more effective carcinogen than B(a)P. The latter was found to commonly bind to guanine residues on DNA because it is less effective in tumor initiation than hydrocarbon-deoxyguanosine adducts [55]. Metabolites from DMBA under the activation of CYP1B1 have been shown to play a vital role in different tumors, where there are elevated levels of CYP1B1 compared to levels of normal nearby tissues, which include the breast, brain, ovary, colon, and lung [55] (Figure 2). In addition to its activity toward exogenous compounds, CYP1B1 is also responsible for the metabolism of endogenous substrates such as estrogen into reactive metabolites, e.g., 4-hydroxyestradiol (Table 1). Estrogen affects the carcinogenesis of endometrial and breast tissues by acting both as an initiator and a proliferator.

#### 2.1.2. P450s and nitrosamine 4-(methylnitrosamino)-1-(3-pyridyl)-1-butanone (NNK)

Nitrosamine 4-(methylnitrosamino)-1-(3-pyridyl)-1-butanone (NNK) is bioactivated in human lung cancer by CYP2A13. In this respect, the enzyme is more effective than CYP2A6. The Vmax/Km is 0.008 for NNK methylene hydroxylation, which is thought to be a significant step in ultimate carcinogen formation by CYP2A6. By comparison, that for CYP2A13 is 0.36 [56]. This elevated level of CYP2A13 bioactivation and the expression of CYP2A13 in the human liver indicate that CYP2A13 could play a major part in NNK activation, which is more effective in CYP2A13 in vitro metabolism than CYP2A6 [57]. Many different CYP2A subfamily members can effectively activate several carcinogenic nitrosamines in vitro [58]. These P450s play a significant role in NNK metabolic activation in vivo in the human liver and mouse lung. Studies also suggest that some P450 enzymes belonging to subfamilies play an important role in the metabolism of another tobacco-specific nitrosamine, N′-nitrosonornicotine, in both humans and rats [59–62]. Approximately 1 to 10% of the total P450 content in the human liver is composed of CYP2A6, which is a particularly efficient coumarin 7-hydroxylase (19). CYP2A6 is the primary enzyme responsible for nicotine metabolism in smokers, and the inactive metabolite in vivo is cotinine [11, 63]. CYP2A13 has approximately 94% sequence similarity to CYP2A6 and differs by just 32 amino acids [64]. Originally, CYP2A13 was cloned from the nasal mucosa of humans. The functional enzyme participates in the metabolism of several CYP2A6 substrates, such as NNK, coumarin, and N,N-dimethylaniline. However, the efficiencies of the two enzymes in affecting metabolism are significantly different [65]. NNK is particularly considered important for the formation of human lung cancer and is metabolized more effectively to potentially carcinogenic intermediates by CYP2A13 instead of CYP2A6. Nicotine metabolism by the two enzymes differs with the metabolism of cotinine. Despite the similarity in product distribution, the Km of nicotine 5′-oxidation by CYP2A6 is six times higher than that for CYP2A13 [66]. The CYP2 family plays a pivotal role in the activation and inactivation of precarcinogens. Both genetic and environmental factors result in interindividual differences in the actions driven by P450s.

## 3. Role of P450s in Cancer Metastasis

**Figure 3:**
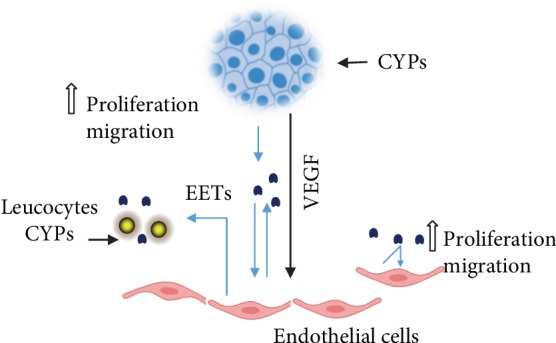
P450s in metastasis and the EET pathways. Blue depicts the crosstalk between the tumor epithelium, endothelial cells, and lymphocytes. This figure was adapted from Edson and Rettie [89].

Metastasis is responsible for most malignant growth-related fatalities, yet it remains the least understood aspect of cancer biology. As metastasis study continues to evolve at a rapid pace, the biological mechanisms underlying the spread and metastatic output of malignant growth cells are beginning to be elucidated [67]. The P450s show promising results in the treatment of cancer due to varying epoxygenases, their impact in cancer progression, and their various expression patterns. EET therapy significantly increased the profiles of migration, invasion, and prometastatic gene expression in a range of cancers. P450 epoxygenases result in the conversion of arachidonic acid into four regioisomeric epoxyeicosatrienoic acids (REA) [68]. These enzymes exert different biological responses in different systems. Several studies found overexpression of CYP2J2 epoxygenase in human cancer cell lines and tissues and increased tumor growth due to EETs, reduced apoptosis of cancer cells, and enhanced proliferation of carcinoma cells [69–71] (Figure 3). The overexpression of CYP2J2 reduces the endothelial cell adhesion molecule expression induced by cytokines and prevents the adhesion of leukocytes to vessel walls [70]. The effects include suppression of I*κ*B and NF-*κ*B kinase, suggesting anti-inflammatory effects of EETs that are independent of the hyperpolarization of membranes. According to Ma et al., EETs are involved in the upregulation of endothelial nitric oxide synthase and the stimulation of the proliferation and angiogenesis of endothelial cells through the activation of the phosphatidylinositol 3-kinase (PI3K)/Akt and mitogen-activated protein kinase (MAPK) signaling pathways [72]. Addition of synthetic CYP2J2 or EET overexpression protected endothelial cells against injury due to hypoxia-reoxygenation in vitro and showed a fibrinolytic influence by enhancing the expression and activity of the tissue plasminogen activator (t-PA). Moreover, this treatment also prevents dysfunction of postischemic myocardial activity by stimulating the MAPK pathway [70]. These findings show that EETs derived from CYP2J2 play an important role in cardiovascular protection. Still, one recent study found potentially harmful effects of CYP2J2 expression and biosynthesis of EET [70]. CYP2J2 protein and mRNA levels were found to be high in certain human cancer tissues and human-derived cancer cell lines. However, they were not present in adjacent normal tissues and noncancer cell lines [73]. Hence, a number of specific CYP2J2 inhibitors have been developed, and their efficacy in inhibiting tumor progression has been actively studied. CYP2J2 inhibitors such as C26 (1-[4-(vinyl)phenyl]-4-[4-(diphenyl-hydroxymethyl)-piperidinyl]-butanone hydrochloride) caused a marked reduction in tumor proliferation and migration as well as promoted apoptosis in cancer cells [74].

The addition of recombinant adeno-associated viral vector- (rAAV-) mediated delivery of CYP2J2, exogenous EETs, or a selective 14,15-EET epoxygenase known as CYP102 F87V resulted in a high proliferation of cancer cells in vivo and in vitro [75]. The addition of EET or epoxygenase overexpression in neoplastic cell lines resulted in increased activation of the PI3K/Akt and MAPK pathways and increased phosphorylation of the epidermal growth factor receptor (EGFR). Carcinoma cell apoptosis was repressed by the upregulation of the antiapoptotic proteins Bcl-2 and Bcl-xL and downregulation of the proapoptotic protein Bax [76]. The results showed that the epoxygenase activity of P450s plays an important role in the neoplastic phenotype promotion as well as in the pathogenesis of different forms of human cancers.

CYP *ω*-hydroxylases, which mainly consist of CYP4F and CYP4A, promote the metabolism of arachidonic acid and the subsequent conversion to biologically active eicosanoids, such as 20-hydroxyeicosatetraenoic acid (20-HETE), which has different pathological and physiological functions [77, 78]. This molecule was found to serve as an additional messenger in the mitogenic-induced growth factor pathway and as an effective mediator in angiogenesis of vessel sprouting and vascular endothelial growth factor (VEGF). A selective inhibitor of 20-HETE synthesis, N-hydroxy-N-(4-butyl-2 methyl phenyl)-formamidine (HET0016), has been found to prevent the angiogenic responses to VEGF, FGF, EGF, and electrical stimulation in rats [79] [80] and inhibits angiogenesis in the cornea that is stimulated by the proliferation of human U251 glioblastoma cells in vitro [81]. The growth of renal adenocarcinoma in nude mice was inhibited by an antagonist of 20-HETE called WIT002 [82]. Moreover, the cell proliferation of U251 cells increased with the introduction of CYP4A1 via infection in vitro [83, 84]. In contrast, a stable 20-HETE agonist, WIT003, stimulated the cell proliferation of endothelial cells and VEGF expression in vitro [84]. CYP *ω*-hydroxylase affects tumor growth, metastasis, and angiogenesis [78].

Signaling pathways, including MAPK and PI3K/Akt, are considered important in invasion, proliferation, metastasis, and angiogenesis [85, 86]. Recent studies have suggested that CYP *ω*-hydroxylase-derived 20-HETE plays an important role in the activation of PI3K/Akt and ERK1/2 in endothelial cells by affecting cellular functions such as apoptosis [87] and proliferation [88]. Moreover, in 251 human gliomas, CYP4A1-20-HETE gene expression changed cell growth through a mechanism that involves ERK1/2 activation. According to Yu et al., CYP4A11 overexpression can result in an increase in phospho-Akt in A549 cells. Moreover, the overexpression resulted in WIT002 or HET0016 inhibition of endogenous 20-HETE stimulation of PI3K/Akt and ERK1/2. Furthermore, inhibitors of PI3K/Akt (wortmannin) and ERK1/2 (U0126) suppressed WIT003-induced MMP-9 and VEGF expression, which indicates that MMP-9 and VEGF induction by CYP *ω*-hydroxylase involves the ERK1/2 and PI3K signaling pathways [78]. Hydroxylation of AA by CYP4 enzymes such as CYP4F2, CYP4A11, and CYP4F3B at the omega position results in the formation of 20-HETE. This enzyme has been shown to have an effect on tumor angiogenesis and progression and inflammatory processes that are associated with metastasis and tumor growth [77, 89].

The role of CYP AA epoxygenase enzymes as either passengers or drivers of the carcinogenesis process is unknown due to a lack of genetic evidence implicating the enzymes in the natural history or the oncogenic transformation of specific tumor types. The evidence of CYP AA epoxygenase enzymes in cancer comes from research involving genetic analyses [90]. Primary tumor growth and metastasis are enhanced by the promotion of CYP AA epoxygenase enzymes as well as escape from dormancy [90]. Mammary tumor engraftment is inhibited through the knockdown of cancer cell intrinsic CYP3A4 [91]. The results derived through genetic approaches show the supporting role of CYP AA epoxygenase enzymes in the progression and metastasis of tumors, which can serve as an important strategy in cancer therapy. This view is supported by knockout and knockdown research on the tumor or the immediate microenvironment that shows the role of P450s in the spread of multiple types of tumors [74, 92]. Targeted therapies have shown restricted success in reducing the existing metastatic development and enhancing survival when used as monotherapies or combination therapies. There are, however, still minimal and contentious epidemiological data on this significant point, partially owing to the inherent problems in conducting epidemiological surveys of cancer metastasis CYPs. Nevertheless, future studies investigating the relationship between P450s and metastasis will shed more light on this very important aspect of cancer.

## 4. Role of P450s in Chemoprevention

Chemoprevention refers to the administration of a medication for the purpose of preventing disease. Cancer chemoprevention has long been acknowledged as a significant prophylactic approach for reducing the health care system's burden of cancer. Chemoprevention of cancer includes the use of one or more pharmacologically active agents to block, suppress, deter, or reverse invasive cancer growth. Nevertheless, comparatively little research has been performed to characterize the capacity of putative chemopreventive agents to modulate the expression of P450s or to comprehend the relationship between P450s and chemopreventive agents. P450s accelerate the process of activation of compounds to toxic products [44]. Moreover, they participate in many different functions, such as the oxidation of fatty acids and steroid hormones [44]. The modulation of enzyme expression can affect chemical toxicity, mutagenicity, and carcinogenicity. An interesting application of cancer chemoprevention is through the administration of a dietary nontoxic component that helps inhibit or prevent neoplastic disease. The chemopreventive agents are regulated by AhR and are known to be procarcinogenic PAHs. While the CYP1 family members are expressed in extrahepatic tissues, CYP1B1 is different due to it being overexpressed in different tumor types compared to expression in normal tissues [93]. This finding has attracted increased interest. Studies have found that this enzyme affects tumorigenesis due to its ability to activate different carcinogens in the PAH chemical classes, aromatic amines, heterocyclic amines, and nitropolycyclic hydrocarbons. In addition, recent studies have found a link between CYP1B1 polymorphisms and a reduced or enhanced risk of certain types of cancers [94, 95]. CYP1B1 and CYP1A1 may also affect the formation of advanced carcinoma and affect enzymes involved in the metabolism of chemotherapeutic agents that may help prevent tumor cytotoxicity [74]. The most important role played by CYP1B1 is in the metabolism of estradiol. The enzyme catalyzes the hydroxylation of estradiol mainly at the C-4 position. Moreover, C-2 hydroxylation can occur mainly through CYP3A4 and CYP1A2 [96, 97]. However, the preferred pathway outside the liver is C-4 hydroxylation, which may play a part in tumorigenesis induced by estrogen-related factors [98]. The reason for this is the strong ER agonist ability of 4-hydroxyestradiol and the binding affinity for the estrogen receptor that is approximately 1.5 times greater than that of estradiol [99]. Another reason is the subsequent conversion of 4-hydroxyestradiol to estradiol 3,4-quinone, which has been shown to bind with DNA and results in the formation of unstable adducts that cause gene mutations [100]. Researchers studying the effects of CYP1B1 knockout as well as CYP1A1 and CYP1A2 found that animals that lack these genes did not suffer from any deficiencies and displayed normal growth. Moreover, the CYP1B1 knockout mice displayed strong resistance to the formation of tumors induced by DMBA [101]. The studies show proof of the potential safety and efficacy of chemopreventive agents that inhibit the activity and expression of CYP1B1. The ability to induce CYP1 family expression by PAHs is illustrated by studies that show high levels of CYP1B1 and CYP1A1 expression in urothelial and lung tissue of smokers [79]. A practical chemopreventive strategy is treatment with AhR antagonists. The activation of different carcinogens that stimulate CYP1 expression through AhR is possible in the presence of CYP1B1 and CYP1A1 expressions.

### 4.1. Natural Compounds

**Figure 4:**
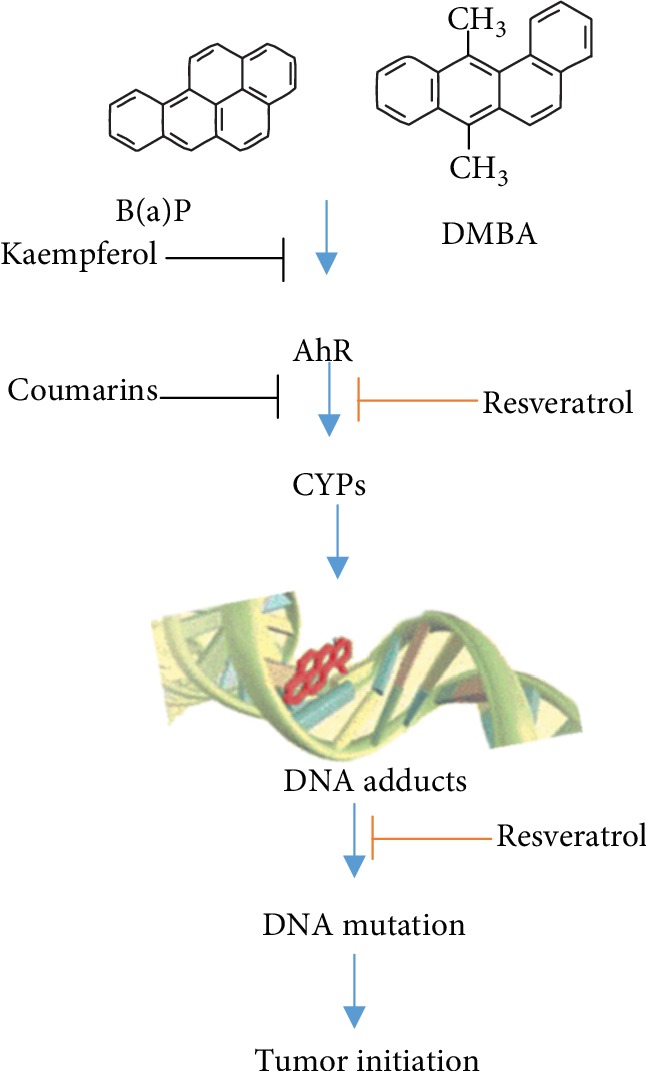
P450s in chemoprevention.

Researchers have found many different natural products showing promising results in this respect. One recent study has shown that the flavonoid kaempferol prevents agonist binding to AhR. Moreover, researchers have found that the compound plays a role in the complex induction and formation of CYP1A1 expression [102, 103] (Figure 4). Another major discovery was that kaempferol prevented the growth of immortalized lung epithelial cells (BEAS-2B) caused by cigarette smoke condensate in a soft agar colony assay. This study found that the compound prevented AhR binding with an IC_50_ of 28 nM at a cellular concentration of 10 *μ*M (~IC90) [102]. Other natural compounds that were found to prevent CYP1 family expression include the stilbene phytoestrogen resveratrol and the *5*,*7-dimethoxyflavone* that prevents CYP1A1 expression and action. Many different compounds have been investigated to assess their ability to inhibit the enzymatic activity of CYP1B1 [35, 104]. Some of the molecules that were found to have potent inhibitory effects include coumarins, resveratrol, stilbenes, and flavonoids. Different compounds that inhibit the CYP1 family are provided in Figure 4. However, it remains to be seen whether CYP1 inhibition results in chemoprevention in vivo.

An alternative CYP1-based chemotherapy technique involves the introduction of an inactive prodrug to a cytotoxic compound. Studies have found that resveratrol can be metabolized to piceatannol through CYP1B1 within cancerous cells [105]. Different synthetic drugs that are specifically activated by CYP1 agents have been developed. One compound that shows promising results is phortress, which is a benzothiazole prodrug that has begun phase I clinical trials [106]. The hydrophilic lysine-amide compound does not undergo hydrolysis of the parent compound 5F203 except in the presence of cells [107]. This compound is then absorbed by sensitive cells and acts as a potent agonist of AhR, resulting in the induction of CYP1 family genes. Subsequently, CYP1A1 metabolizes 5F203, resulting in the production of reactive electrophilic species that cause cell death and DNA damage. The drug has shown strong benefits in preclinical trials both in vivo and in vitro against different types of tumors [108]. Since only AhR-expressing cancer cells are vulnerable to phortress, patients need to be properly screened to ensure that they will benefit from the drug. Recently, phase I clinical trials of aminoflavone (5-amino-2,3-fluorophenyl-6,8-difluoro-7-methyl-4H-1-benzopyran-4-1) have been completed, which is believed to function in a similar manner to 5F203. However, recent studies have shown that aminoflavone requires sulfotransferase A1 (SULTA1) expression for a cellular response [109].

This finding suggests that the activation of the aminoflavone may be complex, and the long-term effects of treating patients with aminoflavone or phortress are unclear. As mentioned earlier, induction of CYP1 family proteins and the activation of AhR will likely lead to the development of cancer and may do more harm in the long run. Nevertheless, the developers of phortress have highlighted the fact that the cell-specific activation of AhR by the drug is different than that of other carcinogens, such as PAHs. Preclinical studies have shown low toxicity levels, but further research is required to prove the clinical effectiveness of aminoflavone and phortress in cancer treatment [110]. An advantage of using a prodrug that is specifically designed to activate tumor-specific CYP1B1 is that it does not induce CYP1 enzymes. An example is DMU-135 (3,4-methylenedioxy-3′,4′,5′-trimethoxy chalcone) that is converted within tumors by CYP1B1 to form DMU-117, which is an effective nonselective tyrosine kinase inhibitor and a possible COX inhibitor [111]. Researchers have found that DMU-135 can act as a chemopreventive agent to prevent the formation of gastrointestinal tumors in mice without toxicity. It is the first drug that is specifically targeted for CYP1B1 activation [111]. Moreover, different CYP2 family members have been found in extrahepatic tissue, of which, the notable ones discovered by the Human Genome Project include CYP2R1, CYP2S1, and CYP2U1 [112]. These enzymes can produce important xenobiotic and endogenous metabolites. For instance, CYP2S1 and CYP2R1 can be used in the metabolism of ATRA and vitamin D hydroxylase, respectively [112]. However, the exact role of the enzymes in tumor progression is not clear. CYP2W1 has been identified in tumor-specific CYP24, particularly adrenal and gastric cancers [113]. Moreover, indole and arachidonic acid have recently been identified as potential substrates. Despite the lack of knowledge on the enzyme functions, the tumor-specific enzyme actions remain intriguing and require further research.

The roles of other P450 family members in vivo suggest their potential use in chemopreventive therapies specifically targeting P450 inhibition [114]. Selective P450 inhibitors, such as methoxylated or hydroxylated flavonols and flavones, methoxytrans-stilbenes, kaempferol, and berberine and rutaecarpine alkaloid derivatives, also serve as promising chemopreventive agents for both estrogen-related and environmental carcinogen-induced carcinogenesis to inhibit the formation of tumors in cancerous cells. In cancer prevention and treatment, targeting P450s with natural or synthetic small molecules provides potential benefits. Since the crystal structures are still to be determined for almost all CYPs, drug design strategies depend on the knowledge of the substrate structure and the mechanism of action of the enzyme. These enzyme-targeting techniques include the following: (i) designing enzyme-inhibiting molecules, (ii) the development of enzyme-activated prodrugs, (iii) immuno-based therapies targeting enzyme immune responses, and (iv) genetic therapy strategies to express different P450s in cancer cells.

## 5. Role of P450s in Chemotherapy

Chemotherapy with cytotoxic antineoplastics remains an important technique for the clinical treatment of patients with malignant tumors. The most important site for P450 metabolism is the liver, where the enzymes are omnipresent. Anticancer drugs are usually metabolized by a number of parallel and/or sequential reactions after absorption in the organism. Metabolism occurred in two distinct sequential phases called “Phase I” and “Phase II,” although this order is not exclusive (Phase I did not always accompany Phase II; Phase II was not always followed by Phase I) [115]. P450s are key players within the phase I-dependent metabolism and for the most part, catalyze the oxidations of drugs [116]. CYP1, CYP2, and CYP3 family members are involved in drug metabolism. P450s catalyze a number of different reactions, including hydroxylation, epoxidation, dealkylation, and deamination, of which hydroxylation is likely the most important. Phase II-dependent reactions make the products suitable for excretion through the kidneys as inactive polar products. With regard to anticancer agents, P450s are involved not only in the detoxification of cytotoxic products but also in the activation of drugs that make them therapeutically active. P450s metabolize many anticancer agents, shown in Table 2. Prodrugs are inactive agents that, upon exposure to tumor tissues, are converted to active cytotoxic drugs with high expression of activating enzymes. This targeting strategy minimizes toxicity to normal tissues while increasing the tumor tissue delivery of the active agent. Liver P450s metabolize cyclophosphamide, ifosfamide, dacarbazine, procarbazine, tegafur, and thiotepa [117]. Another example is 1,4-bis-([2-(dimethylamino-N-oxide)ethyl]amino)5,8-dihydroxy anthracene-9,10-dione (AQ4N), a bioreductive drug that requires CYP2S1 and CYP2W1 activation in tumor tissues to be transformed into an inhibitor of topoisomerase II [118].

The use of immunohistochemistry, western blot analysis, and reverse transcriptase PCR has shown reliable expression of CYP3A types in kidney cancer cells. This study suggested that the expressed CYP3A may be involved in the development of renal cancer and that the multidrug resistance found in this cancer is caused by these types of CYP3A [119, 120]. CYP3A4 plays a major role in the metabolism of several anticancer agents (taxanes, vinca-alkaloids, and new drugs such as imatinib, gefitinib, and sorafenib). CYP3A4 metabolizes docetaxel into hydroxylated derivatives that are inactive. A high activity of CYP3A4 will result in the drug's poor therapeutic outcome. Thus, a 49% decrease in docetaxel clearance was observed in patients with cancer treated with docetaxel in conjunction with the active CYP3A4 inhibitor ketoconazole [121]. Low CYP3A4 expression in breast tumors improved the response to docetaxel. Likewise, hepatic CYP3A4 activity assessed by the erythromycin breath test and midazolam clearance predicted the clearance of docetaxel and showed lower toxicity in patients with the lowest CYP3A4 activity. Unlike docetaxel, irinotecan is inactivated by CYP3A4, and the induction of CYP3A4 results in a significant decrease in the development of the toxic metabolite of this drug in patients receiving irinotecan. In addition, the CYP3A4 phenotype is significantly associated with irinotecan pharmacokinetics as measured by midazolam clearance. A study recently indicated that the pregnane X-receptor (PXR) pathway also includes irinotecan resistance in the colon cancer cell line through the upregulation of drug-metabolizing genes such as CYP3A4 [122–124].

The main expression pattern of CYP4Z1 renders it a suitable candidate for cancer therapy. However, for prodrug therapy based on CYP4Z1 for the treatment of breast cancer, lung cancer, ovarian cancer, and prostate cancer, effective prodrugs must be identified. Previous studies have used CYP enzymes to activate different anticancer prodrugs [125]. Most researchers have investigated oxazaphosphorines, including ifosfamide and cyclophosphamide [126–128]. In clinical trials, CYP2B6-dependent activation of cyclophosphamide has shown positive results [129, 130]. Presently, researchers are working on new mutants of CYP2B6 to improve the activation of cyclophosphamide. For instance, Haque and Pattanayak found a superior ability of a triple mutant of CYP2B6 to fuse with NADPH cytochrome P450 reductase that can help in converting cyclophosphamide into its cytotoxic form [131].

Moreover, increasing the number of cancerous tissues results in low P450 activity compared to that of the nearby healthy tissues where the prodrugs are activated, resulting in cytotoxicity. An important cancer research objective is to develop therapeutic agents that specifically target tumor cells. Studies have found that P450s provide therapeutic options at higher levels in tumor cells compared to that in the surrounding tissues due to activation of the prodrugs in the cancer cells, which reduces side effects [20, 132]. As a result, there are opportunities for enhanced tumor-specific endogenous expression of P450s and CYP-mediated gene therapy. Most studies have extensively investigated CYP1B1 as opposed to other P450 family members. The enzymes have shown high expression, particularly in liver cells. CYP1B1 protein expression has been found in different tumors, and the protein was not detected in normal tissues [133]. Many different agents, such as phortress and resveratrol, are activated by CYP1B1 in preclinical studies. Moreover, a CYP1B1 vaccine known as Zyc300 is presently in phase I and II trials that aim to destroy cancer cells through T-cell response induction [117, 134]. These strategies can be initiated with other P450s that have been identified in tumor cells, such as CYP2J2, CYP2W1, and CYP4Z1. This requires the identification of an appropriate prodrug. However, additional research is required to ensure the success of CYP-based cancer therapy.

### 5.1. P450 Polymorphisms

Genetic polymorphisms of P450s can affect the catalytic activity of the enzyme and have been reported to be associated with different diseases and adverse drug reactions in different populations. In terms of drug metabolism, P450 polymorphism phenotypes vary from ultra-fast to weak metabolizers. Of the 50 known P450 isoenzymes catalyzing drug metabolism, more than 20 P450 genes are functionally polymorphic, such as CYP2A6, CYP2C9, CYP2C19, CYP2D6, CYP1B1, and CYP1A2. The polymorphic P450s catalyze about 40% of the drug metabolism [119, 135]. In addition, P450 polymorphisms were reported to confer susceptibility to disease and disease protection or reduced risk (Table 3). Inhibition of P450 enzymes by a new chemical entity (NCE) may decrease the metabolism of comedicated drugs. However, P450s were largely overlooked in the development of cancer drugs until recently, recognized only for their role in the chemotherapeutic metabolism of Phase I. The first successful strategy in cancer therapy to target P450s was the production of active CYP19 (aromatase) inhibitors for the treatment of breast cancer. Aromatase inhibitors have entered a new era in hormone ablation therapy for estrogen-dependent cancers, paving the way for similar strategies to combat androgen-dependent prostate cancer. Identification of the P450s involved in the inactivation of vitamin D3 and vitamin A anticancer metabolites has also triggered the development of agents targeting these enzymes. The discovery in cancer cells of the overexpression of exogenous metabolizing P450s, such as CYP1B1, has increased interest in the creation of chemoprevention inhibitors and prodrugs intended to be triggered by P450s in cancer cells only [74].

Currently, decisions on which drugs to prescribe are made for many disorders on a trial-and-error basis. Genetically based screening methods would allow the tailoring of drug therapy, drug selection, and dosing according to the ability of an individual to metabolize a drug under the pharmacogenomic paradigm. The fact that two genomes are involved complicates cancer pharmacogenomics: the patient's germline genome and the tumor's somatic genome. Chemotherapeutic drugs are highly sensitive to genetic background, as they are generally unspecific drugs with narrow therapeutic indexes that often result in severe or fatal toxicity.

#### 5.1.1. Cyclophosphamide

Cyclophosphamide (CPA), a prodrug used in cancer therapy, is activated by CYP2C19, CYP2C9, CYP3A4, and CYP2B6 to treat some autoimmune disorders. CYP2C19∗2 and CYP2B6∗5 carriers have been shown to have a significantly lower removal of CPA and worse therapeutic performance. Also in the liver, CYP2B6 enzyme metabolizes ifosfamide, tamoxifen, procarbazine, and thiotepa in the same way as it activates CPA [117].

#### 5.1.2. Tamoxifen

Tamoxifen is a modulator of estrogen receptors used in hormone receptor-positive breast cancers. It has been suggested that CYP2D6, the active metabolizer of tamoxifen, is necessary for the formation of endoxifen. Several studies have shown that CYP2D6 PMs have decreased relapse-free time and disease-free survival rate, but they do not experience hot flashes of the same magnitude as patients with the wild-type allele. As a result of enzyme inhibition (serotonin reuptake inhibitors, antidepressants, and other inhibitors of CYP2D6), a similar loss of efficacy is observed [117].

#### 5.1.3. Thalidomide

Thalidomide bioactivation depends on CYP2C19 (5-hydroxythalidomide) metabolism. Another pathway also exists that produces thalidomide arene oxide and is mediated by CYP1A1 and CYP2E1. The response to thalidomide and dexamethasone parallel treatment was reported to be higher in CYP2C19 EMs than in PMs in multiple myeloma. The lower response rate observed in PMs may be due to reduced angiogenesis inhibition activity. Notwithstanding this evidence, the CYP2C19 polymorphism does not have a major influence on the treatment outcome [136].

#### 5.1.4. Tegafur

Tegafur is a drug that is converted by CYP2A6 to 5-fluorouracil. The drug is weakly metabolized in patients with CYP2A6∗4 or CYP2A6∗11. Since other P450s influence tegafur metabolism (CYP3A4, CYP3A5, glutathione S-transferases), it is difficult to calculate the effective dose [137].

#### 5.1.5. Imatinib

Imatinib mesylate (IM), a specific inhibitor of the BCR-ABL tyrosine kinase, is a well-established first-line treatment for chronic myeloid leukemia (CML). IM is utilized for the most part by P450s in the liver, specifically the CYP3A4 and CYP3A5 catalysts. Polymorphisms in these genes can modify the protein action of IM and may influence its reaction. Yuan et al. reported the effect of two single-nucleotide polymorphisms (SNPs), namely, CYP3A5∗3 (6986A>G) and CYP3A4∗18 (878T>C), on IM treatment reaction in patients with CML (*n* = 270; 139 IM resistant and 131 IM responsive) [138].

## 6. Conclusion

P450s play a vital role in chemoprevention, carcinogenesis, cancer therapy, and metastasis through regulation. As a result, whether inhibition of P450s reduces the risk of cancer depends on the cancer type, its etiology, and the treatment. The literature review shows that much progress has been made in understanding the role of drug-metabolizing P450s in cancer treatment. The P450 family genes may play a role in the formation of different types of cancer, as demonstrated by their overexpression, which promotes carcinogenicity. Certain P450 family members are upregulated in cancer, making them potential targets for cancer treatment. Thus, by providing P450-mediated metabolism at the tumor site such as the site of anticancer drug action, individual P450s, which are overexpressed in tumor cells, may represent exciting and novel targets for cancer. In addition, patient-specific therapeutic regimens, including prodrugs, reversible inhibitors, and immunotherapy, can be customized to facilitate the management of a variety of human tumors by recognizing the complement of functionally active P450s within the tumor and nontumor tissues. However, whether the enzyme activities need to be inhibited or enhanced depends on different types of cancer and the important products that these P450s produce. Recent accomplishments in the use of polymorphic C as drug targets in cancer therapy are promising and could provide a new and effective alternative for future cancer therapy.

## Figures and Tables

**Table 1:** P450s involved in the bioactivation of chemical carcinogens.

Types	Carcinogens	Compound	Ref
CYP1A1 CYP1A2 CYP1B1	PAH, arylamine, heterocyclic amine, nitroarene, and estrogen	Benzo[a]pyrene (B[a]P); 7,12-dimethylbenz[a]anthracene (7,12-DMBA); benz[a]anthracene (B[a]A); benzo[c]phenanthrene; 5-methylchrysene; dibenzo[a,l]pyrene (DB[a,l]P); 3-methylcholanthrene (3-MC); fluoranthene; 2-Acetylaminofluorene; 2-amino-6-methyldipyrido[1,2-a: 3′,2′-d]imidazole (Glu-P-1); 3-amino-1,4-dimethyl-5H-pyrido[4,3-b]indole (Trp-P-1); 2-amino-1-methyl-6-phenylimidazo[4,5-b]pyridine (PhIP); 17*β*-estradiol; estrone; 4-hydroxyestradiol; 1-Nitropyrene; 2-nitropyrene; 6-nitrochrysene; 2-aminofluorene; 2-aminoanthracene; 6-aminochrysene; 2-Amino-3-methylimidazo[4,5-f]quinoline (IQ); 2-amino-3,5-dimethylimidazo[4,5-f]quinoline (MeIQ)	[131–136]
CYP2A6	Mycotoxin, alkenylbenzene: occurs in a variety of foods including essential oils of tarragon, sweet basil, sweet fennel, tobacco-specific nitrosamine, and nitrosamine	1, 2-Dibromoethane (ethylene dibromide); 1,3-butadiene, 2,6-dichlorobenzonitrile (dichlobenil); 3-(N-nitrosomethylamino) propiona aldehyde; benzhydrol; butadiene monoxide (1,2-epoxy-3-butene, methoxsalen); (8-methoxypsoralen, xanthotoxin), N-nitrosomethylbutylamine; N-nitrosopiperidine; N-nitrosopyrrolidine; p-benzoylphenol (4-hydroxybenzophenone)	[134, 137–139]
CYP2A13	*Nitrosamin*e	4-(Methylnitrosamino)-1-(3-pyridyl)-1-butanone (NNK); N-nitrosonornicotine (NNN); 3-(*N*-nitrosomethylamino) propionaldehyde; 3-methylindole (skatole); 3-N-nitrosoguvacoline; 4-(methylnitrosamino)-1-(3-pyridyl)-1-butanone (NNK); bergapten, 5 methoxypsoralen; N-nitrosopyrrolidine	[58, 140–142]
CYP3A4	Difuranocoumarin, mycotoxin	Aflatoxin B1; aflatoxinG1; sterigmatocystin; dihydrodiol derivatives of PAHs	[143–146]
CYP2B6	Haloalkane, azoaromatic amine, tobacco-specific nitrosamine, herbicide, chloroacetamide, PAH, hydrocarbon, and alkyl benzene	1, 2-Dibromoethane (ethylene dibromide); 2, 2-dichloro-1,1,1-trifluoroethane (HCFC-123); 3-methoxy-4-aminoazobenzene; 4-vinyl-1-cyclohexene; (*S*)- and (*R*)-; *N*, *N*′, *N*^″^-triethylene thiophosphoramide (thioTEPA)	[29, 147–150]
CYP2C8	Oxazaphosporine: anticancer, nitrogen mustard alkylating, tobacco-specific nitrosamine, nitrosamine, aromatic hydrocarbons, alkyl benzene	Ifosfamide; 4-(methylnitrosamino)-1-(3-pyridyl)-1-butanone (NNK); chloroform (trichloromethane); N-nitrosomethylpropylamine; styrene	[151–153]
CYP2C9	Aliphatic epoxide, metabolite, oxazaphosphorine: anticancer, nitrogen mustard, alkylating, phenylpropene, from Rhizoma acorigramine, and nitrosamine	Butadiene monoxide (1, 2-epoxy-3-butene); cyclophosphamide; ifosfamide; methyleugenol; *N*-nitrosopyrrolidine	[134, 154–156]
CYP2D6	Nitrosamine, tobacco-specific nitrosamine, difuranocoumarin; mycotoxin, produced by Aspergillus species on food products, pyrido-carbazole; antineoplastic, alkaloid, Apocynaceae plant compound, topoisomerase II inhibitor and DNA binding	3-(*N*-nitrosomethylamino) propionaldehyde; NNK; NNAL; aflatoxin B_1_ (AFB_1_); ellipticine	[157–159]
CYP2E1	Haloalkane, diene halobenzene, nitrile, herbicide, arylamine, and furanoterpene produced in sweet potatoes infected with Fusarium solani; pulmonary toxin, alkylating, organic solvents, alkylformamide, nitrosamine, o-methoxyaniline, cyclohexane derivative	1, 2-Dichloroethane (ethylene dichloride); 1,3-butadiene; 1,4 and 2,3-dichlorobutane; 2,6-dichlorobenzonitrile (dichlobenil); 2-aminofluorene (2-AF); 4-ipomeanol; *N, N*-dimethylformamide (DMF), *N*-nitrosoethylbutylamine; *o*-anisidine 2-methoxyaniline; vinylcyclohexane	[160–164]
CYP2F1	Indole, alkylating, pulmonary toxin; present in higher concentrations in mammalian digestive tract and coal tar, furanoterpene produced in sweet potatoes infected with Fusarium solani; pulmonary toxin, alkylating, aromatic hydrocarbon, alkyl benzene	3-methylindole, skatole; 4-ipomeanol; styrene (vinyl benzene)	[165–167]
CYP2W1	PAH, metabolite	Chrysene-1, 2-diol, dibenzo[*a,l*]pyrene-11,12-diol, sterigmatocystin	[168]
CYP3A4	Nitroarene, triazole, heterocyclic amine, azoaromatic amine, N-heterocyclic aromatic hydrocarbon, dibenzocarbazole, estradiol derivative; estrogen, contraceptive, nitrosamine, triphenylethyleneamine; antiestrogen, estrogen receptor modulator	1-Aminobenzotriazole (1-ABT); 1-aminopyrene; 1-nitropyrene; 2-aminofluorene; 3,6-dinitrobenzo[*e*]pyrene; 3-amino-1,4-dimethyl-5H-pyrido[4,3-*b*]indole (Trp-P-1); 3-methoxy-4-aminoazobenzene; 7*H*-dibenzo[*c*,*g*]carbazole; 17*α*-ethynylestradiol (ethi-nylestradiol 17*α*-); *N*-nitrosodibutylamine (*N*,*N*-dibutylnitrosamine); tamoxifen	[134, 149, 169]
CYP3A5	Antimitotic, epipodophyllotoxin, topoisomerase II inhibitor, oxazaphosphorine; nitrogen mustard alkylating	Etoposide (VP-16); ifosfamide; tobacco-specific nitrosamine	[134, 149]

**Table 2:** P450s involved in cancer drug metabolism.

Drugs	CYPs	Ref
Altretamine	2B	[170]
Bexarotene	2C9, 3A4	[171]
Busulfan	3A4	[172]
Cisplatin	2E1, 3A4	[173]
Cyclophosphamide	2B6, 2C9, 3A4	[174]
Cytarabine	3A4	[175]
Dacarbazine	1A1, 1A2, 2E1	[176, 177]
Docetaxel	1B1, 3A4, 3A5	[176]
Doxorubicin	2D6, 3A4	[178]
Erlotinib	1A1, 1A2, 3A4	[179]
Etoposide	1A2, 2E1, 3A4, 3A5	[180]
Exemestane	3A4	[181]
Fulvestrant	3A4	[182]
Gefitinib	3A4	[183]
Idarubicin	2D6, 2C9	[184]
Ifosfamide	2A6, 2B1, 2B6, 2C9, 2C18, 2C19, 3A4, 3A5	[185]
Imatinib mesylate	1A2, 2C9, 2C19, 2D6, 3A4	[186]
Irinotecan	3A4, 3A5	[187]
Letrozole	2A6, 3A4	[188]
Paclitaxel	2C8, 3A4, 3A5	[189]
Tamoxifen	1A1, 1A2, 1B1, 2B6, 2C9, 2C19, 2D6, 2E1, 3A4, 3A5	[190]
Teniposide	3A4, 3A5	[180]
Thiotepa	2B1, 2C11	[191]
Topotecan	3A4	[192]
Toremifene	1A2, 3A4	[193]
Tretinoin	2C8, 2C9, 2E, 3A4	[194]
Vinblastine	3A4	[195]
Vincristine	3A4	[196]
Vinorelbine	3A4	[197]

**Table 3:** Diseases associated with P450 polymorphism.

S. no.	CYPs	Diseases	Gene polymorphism	Country of population	Ref
1	CYP1A2	Prostate cancer	T3801C at 3′UTR	Indian	[198]
2	CYP7A1	Tuberculosis	rs3808607	Moroccan	[199]
3	CYP1A2	Cancers	rs762551	Caucasians	[200]
4	CYP17A1	Prostate cancer	Wild	General	[201]
5	CYP24A1∗	Idiopathic infantile hypercalcemia	rs114368325 rs6068812	German, Russia, Turkey	[202]
6	CYP8A1	A left main coronary artery disease	C1117	Greece	[203]
7	CYP19A1	Alzheimer disease	rs3751592	Chinese	[204]
8	CYP1B1	T2D	rs1056827	Saudi Arabians	[205]
9	CYP4A11	Hypertension	rs1126742 rs3890011	Chinese	[206]
10	CYP1B1	Atherosclerosis	Wild	mice	[207]
11	CYP2C9	Epistatic interactions to coronary heart disease susceptibility	rs9332242 and rs61886769	Russian	[208]
12	CYP2J2	Ischemic stroke	-50G/T	Chinese	[209]
13	CYP17	Gallbladder cancer /breast cancer	rs743572	Indian/Chinese	[127]

## Abbreviations

P450s: Cytochrome P450s
PAHs: Polycylic aromatic hydrocarbon
EETs: Epoxyeicosatrienoic acids
DHETs: Dihydroxyeicosatrienoic acids
GBMs: Glioblastomas
PHAH: Polyhalogenated aromatic hydrocarbons
AhR: Aryl hydrocarbon receptor
NF-*κ*B: Nuclear factor *κ*B
RB1: Retinoblastoma protein 1
DMBA: 7,12-Dimethylbenz[a]anthracene
B(a)P: Benzo[a]pyrene
NNK: Nitrosamine 4-(methylnitrosamino)-1-(3-pyridyl)-1-butanone
MAPK: Mitogen-activated protein kinase
PI3K: Phosphatidylinositol 3-kinase
EGF: Epidermal growth factor
VEGF: Vascular endothelial growth factor
ARNT: Aryl hydrocarbon nuclear translocator
DMU-135: 3,4-Methylenedioxy-3′,4′,5′-trimethoxy chalcone
ROM: Reactive oxygenate intermediate
NCE: New chemical entity
XME: Xenobiotic-metabolizing enzyme
REA: Regioisomeric epoxyeicosatrienoic acids
t-PA: Tissue plasminogen activator
rAAV: Recombinant adeno-associated viral vector
SULTA1: Sulfotransferase A1
PXR: Pregnane X-receptor
CML: Chronic myeloid leukemia
SNPs: Single-nucleotide polymorphisms
IM: Imatinib mesylate.
